# Blood virome profiling reveals subtype-specific viral signatures and reduced diversity in non-Hodgkin lymphoma

**DOI:** 10.1080/21505594.2025.2542457

**Published:** 2025-08-17

**Authors:** Shaokun Pan, Wang Li, Xingyue Zhao, Huijie Wang, Jing Liu, Wen Zhang, Chenglin Zhou, Youhua Xie

**Affiliations:** a Key Laboratory of Medical Molecular Virology (MOE/NHC/CAMS), Shanghai Institute of Infectious Diseases and Biosecurity, Shanghai Frontiers Science Center of Pathogenic Microbes and Infection, Department of Microbiology and Parasitology, School of Basic Medical Sciences, Shanghai Medical College, Fudan University, Shanghai, China; b Clinical Laboratory Center, The Affiliated Taizhou People’s Hospital of Nanjing Medical University, Taizhou, China; c Department of Laboratory Medicine, School of Medicine, Jiangsu University, Zhenjiang, China; d Department of Clinical Medicine, Clinical Medical College of Anhui Medical University, Hefei, China; e Department of Medical Oncology, Shanghai Cancer Center, Fudan University, Shanghai, China

**Keywords:** Non-Hodgkin lymphoma (NHL), blood virome, viral metagenomic sequencing, *Anelloviridae*, human pegivirus-1 (HPgV-1), Picobirnavirus

## Abstract

Non-Hodgkin lymphoma (NHL), a heterogeneous lymphoid malignancy, demonstrates molecular diversity linked to genetic and immune factors, with emerging roles for viral infections in pathogenesis. Yet, the blood virome’s composition and dynamics in NHL remain poorly characterized. This study characterizes the blood virome in NHL subtypes using viral metagenomic sequencing of serum from 217 patients (B-cell: BCL, T-cell: TCL, NK-cell: NKCL) and 40 healthy controls. Bioinformatic analysis identified 45 viral families, revealing subtype-specific viromic signatures. BCL exhibited a dominance of *Anelloviridae*, which accounted for 86% of eukaryotic viruses, compared with only 3% in controls, correlating with immunosuppression. Additionally, picobirnavirus, an opportunistic pathogen particularly in hosts with compromised immune systems, also showed a significant difference compared to controls. NKCL showed *Flaviviridae* enrichment, accounting for 82% of eukaryotic viruses, with nearly all of them being human pegivirus-1 (HPgV-1). Compared with healthy controls, patients with NHL exhibited significantly lower blood virome α-diversity at the genus level, and T-cell lymphomas showed the lowest species-level richness (140 vs. 332 in controls). Beta diversity highlighted BCL-specific viral heterogeneity, contrasting conserved T/NKCL viral profiles. *Anelloviridae* and Picobirnavirus expansion aligns with immune dysfunction, whereas NKCL-restricted HPgV-1 prevalence underscores biomarker potential. These findings implicate blood virome alterations marked by viral family predominance and diversity loss in NHL pathogenesis via immune modulation or oncogenesis. This first comprehensive NHL virome profile identifies subtype-specific signatures (*Anelloviridae*/Picobirnavirus/HPgV-1) for potential diagnostic and therapeutic targeting. Validation of these biomarkers may refine NHL subtyping and elucidate virome-lymphomagenesis mechanisms.

## Graphical abstract

**Figure:**
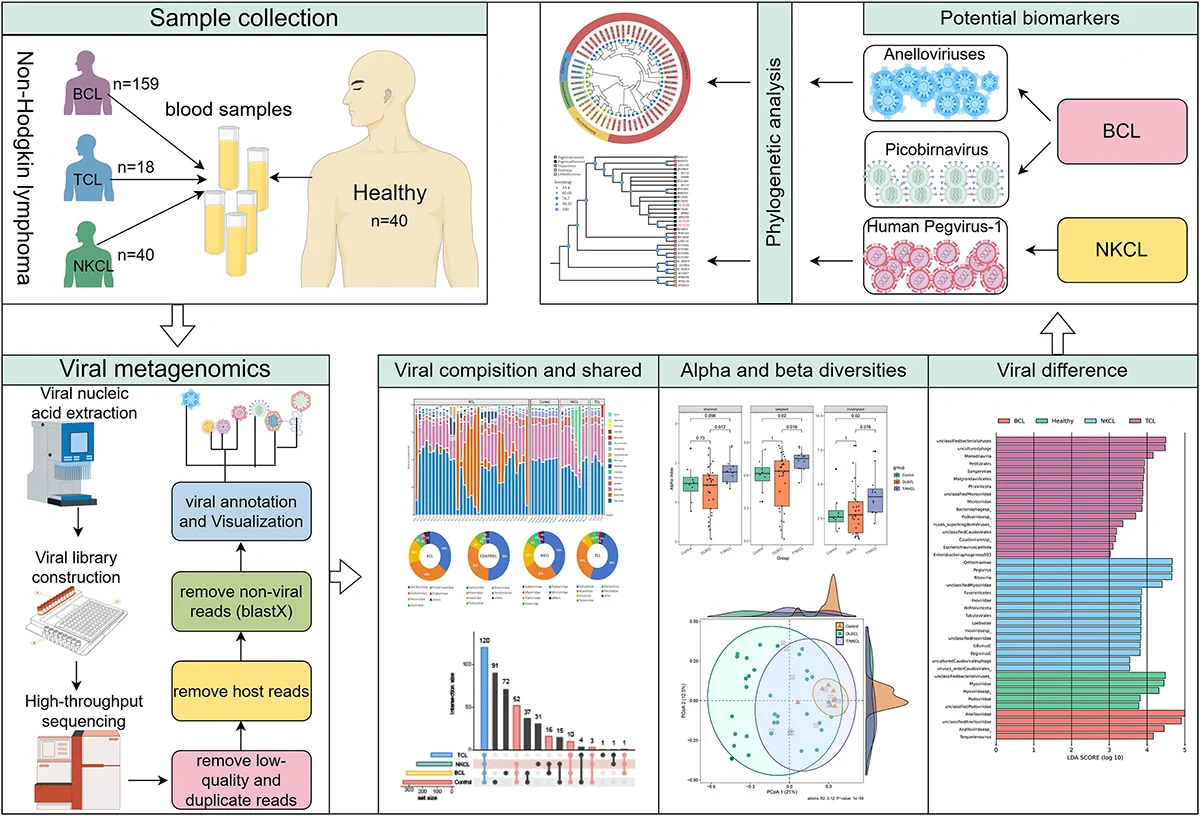

## Introduction

>Lymphoma, a heterogeneous group of malignancies originating from lymphocytes, represents one of the most prevalent haematological cancers worldwide. Classified into Hodgkin lymphoma (HL) and non-Hodgkin lymphoma (NHL), these neoplasms demonstrate distinct clinical-pathological features and molecular profiles [1,2]. According to GLOBOCAN 2020 estimates, lymphoma accounts for approximately 3% of all cancer diagnoses and 2.8% of cancer-related mortality globally [3]. NHL accounts for roughly 85–90 % of all lymphomas [4] and, in the WHO 5th Edition classification of haematolymphoid tumours (WHO-HAEM5) [5], is organized by lymphoid cell lineage into a four-level hierarchy – Category (Level 1), Family/Class (Level 2), Entity/Type (Level 3) and, for certain neoplasms, Subtype (Level 4). In total, NHL comprises > 80 mature B-cell, T-cell and NK/T-cell neoplasms. For example, Diffuse large B-cell lymphoma, NOS (DLBCL) is a Level 3 entity in the Level 2 family “Large B-cell lymphomas” under the Level 1 category “Mature B-cell neoplasms;” Anaplastic large cell lymphoma, ALK-positive (ALCL) is a Level 3 entity in the Level 2 family “Anaplastic large cell lymphoma” within the Level 1 category “Mature T-cell and NK-cell neoplasms;” and Extranodal NK/T-cell lymphoma, nasal type is a Level 3 entity in the Level 2 family “EBV-positive NK/T-cell lymphomas,” also under “Mature T-cell and NK-cell neoplasms.” [6,7] (See WHO-HAEM5 Table 1 and 2 for the complete list of entities.) As one of the most common cancers of the lymphatic system, NHL exhibits significant variability in its clinical presentation, molecular pathology, and response to treatment [5,8]. While genetic mutations, immune dysregulation, and environmental factors are well-established contributors to NHL pathogenesis, emerging evidence suggests that microbial infections, particularly viral infections, may play a critical role in the development and progression of certain NHL subtypes. Viruses such as Epstein-Barr virus (EBV), human T-cell leukaemia virus type 1 (HTLV-1), and hepatitis C virus (HCV) have been directly linked to specific NHL subtypes, including Burkitt lymphoma, adult T-cell leukaemia/lymphoma, and marginal zone lymphoma, respectively [9–11].

The human body harbours a vast and diverse array of viruses, collectively forming a complex virome that plays a critical role in human health and disease [12,13]. Recent advances in high-throughput sequencing technologies have revolutionized the field of microbiome research, enabling comprehensive analysis of microbial communities, including the virome, within the human body [14,15]. Although studies have shown that the gut microbiota may promote the occurrence and progression of cancer including lymphoma by influencing the immune system and chronic inflammation [16–19], research on the blood virome related to non-Hodgkin lymphoma remains scarce. In patients with NHL, it remains unclear whether the blood harbours previously unidentified viruses that may play a role in disease initiation and progression. Additionally, it is critical to investigate whether the blood virome in NHL patients demonstrates significant alterations compared to that of healthy individuals and whether these virome changes are mechanistically associated with NHL development and disease progression.

The primary objective of this study is to characterize the blood virome in NHL patients and to identify viral signatures associated with specific NHL subtypes, and explore the blood virome as a potential source of biomarkers for NHL diagnosis and treatment.

## Materials and methods

### Sample collection and preparation

For viral metagenomic analysis, a total of 217 serum samples were collected from patients diagnosed with non-Hodgkin lymphoma (NHL), comprising 127 males and 90 females, with a mean age of 51.7 ± 14.6 years (range: 14–88 years). Additionally, 40 serum samples were obtained from healthy individuals, including 22 males and 18 females, with an average age of 41.9 ± 10.4 years (range: 19–64 years). All samples were collected at the Department of Clinical Laboratory, Shanghai Cancer Center, between September 2015 and July 2016. Each serum sample was aliquoted into a 2 mL sterile EP tube and immediately stored at −80°C to ensure preservation of viral nucleic acids for subsequent analysis (Supplementary Table S1).

### Viral metagenomic library construction

All the serum samples stored at −80°C were thawed at 4°C and subsequently vortexed at 1,800 rpm for 5 minutes to ensure homogeneity, then centrifuged at 12,000 × g for 15 minutes at 4°C to pellet larger particles, including host cells and other solid debris. The clarified supernatant was transferred to a new microcentrifuge tube for downstream processing. The 217 NHL patient serum samples were classified by lymphoma cell-of-origin immunophenotype (B-cell, T-cell, or NK -cell) and, to ensure adequate viral yield, pooled into 45 groups of four to five sera for library construction; no additional pooling by clinical aggressiveness was performed. Similarly, 40 serum samples from healthy individuals were divided into 8 groups, with each pool containing 5 samples (Supplementary Table S2). For each group above, 50–100 µl equal amount serum was taken from each sample and pooled into one tube. Each pooled supernatant was filtered through a 0.45 μm pore-size filter (Millipore) to remove residual host cells, bacteria, and solid particles [20]. Using a mixture of DNase, RNase, benzonase, and Baseline-ZERO, the filtrates were then treated to degrade free (non-virion-encapsidated) nucleic acids, followed by incubation at 37°C for 60 minutes [21,22]. Total viral nucleic acids were extracted using the QIAamp Viral RNA Extraction Kit (Qiagen), with RNase inhibitor added to prevent RNA degradation. SuperScript III Reverse Transcriptase (Thermo Fisher Scientific) was used with six-base random primers to perform reverse transcription, followed by double-stranded DNA synthesis using Klenow fragment polymerase (New England BioLabs) [23]. Viral libraries were constructed using the Nextera XT DNA Sample Preparation Kit (Illumina) and Nextera XT Index Kit, and paired-end sequencing (250 bp) was conducted on an Illumina MiSeq platform. The sequencing coverage and quality statistics for each library are summarized in Supplementary Table S3.

### Viral metagenomic raw data processing

Paired-end sequencing with a read length of 250 bp was generated by MiSeq sequencing, and using Illumina’s proprietary software to assign reads to their respective DNA libraries. Raw data processing was conducted using a new version in-house analysis pipeline running on a 36-nodes Linux cluster. Adapter trimming, low-quality base filtering, and duplicate removal were performed using fastp software with default parameters. To minimize contamination from host sequences, Bowtie2 was utilized to filter out reads aligning to human and bacterial genomes. Subsequently, non-viral reads were excluded by aligning the cleaned reads to a custom viral proteome database using Diamond software for BLASTx alignment with an E-value cut-off of < 10^−5^. Then the viral paired-end reads were merged and annotated using MEGAN software. Finally, viral reads within each barcode were assembled *de novo* using MEGAHIT to reconstruct viral genomes.

### Viral community analysis

The viromic data of the 53 libraries were normalized using MEGAN 6.22.2 to eliminate the differences caused by varying sequencing depths among the libraries. Viral community analyses were conducted using R version 4.4.1, incorporating packages such as ggplot2, RColorBrewer, vegan, UpSetR, ggpubr and reshape2. Alpha and beta diversities were analysed using the Wilcoxon test. Differences in viral communities between NHL group and healthy group were assessed using STAMP analysis with Welch’s t-test. Additionally, LEfSe analysis was performed using an online tool available at http://galaxy.biobakery.org/.

### Phylogenetic analysis

Phylogenetic analysis of Anelloviriuses was conducted using the putative capsid protein sequences identified in this study, along with reference strains representing diverse genera within the *Anelloviridae* family obtained from GenBank. For *Flaviviridae*, phylogenetic reconstruction was performed based on the polyprotein sequences of pegiviruses identified in the current study, supplemented by reference sequences spanning multiple genera of *Flaviviridae* retrieved from GenBank. The largest viral open reading frames (ORF1s) were predicted and annotated using Geneious Prime v2024.0.5, with manual validation of protein domain boundaries. Sequence alignment was conducted using MUSCLE in MEGA v11.0.13 with default parameters, followed by the construction of maximum likelihood (ML) phylogenetic trees with 1,000 bootstrap replicates to assess node support. The resulting phylogenetic trees were visualized and annotated using the Interactive Tree of Life (iTOL) platform.

## Results

### Viral population composition

**Figure 1.**
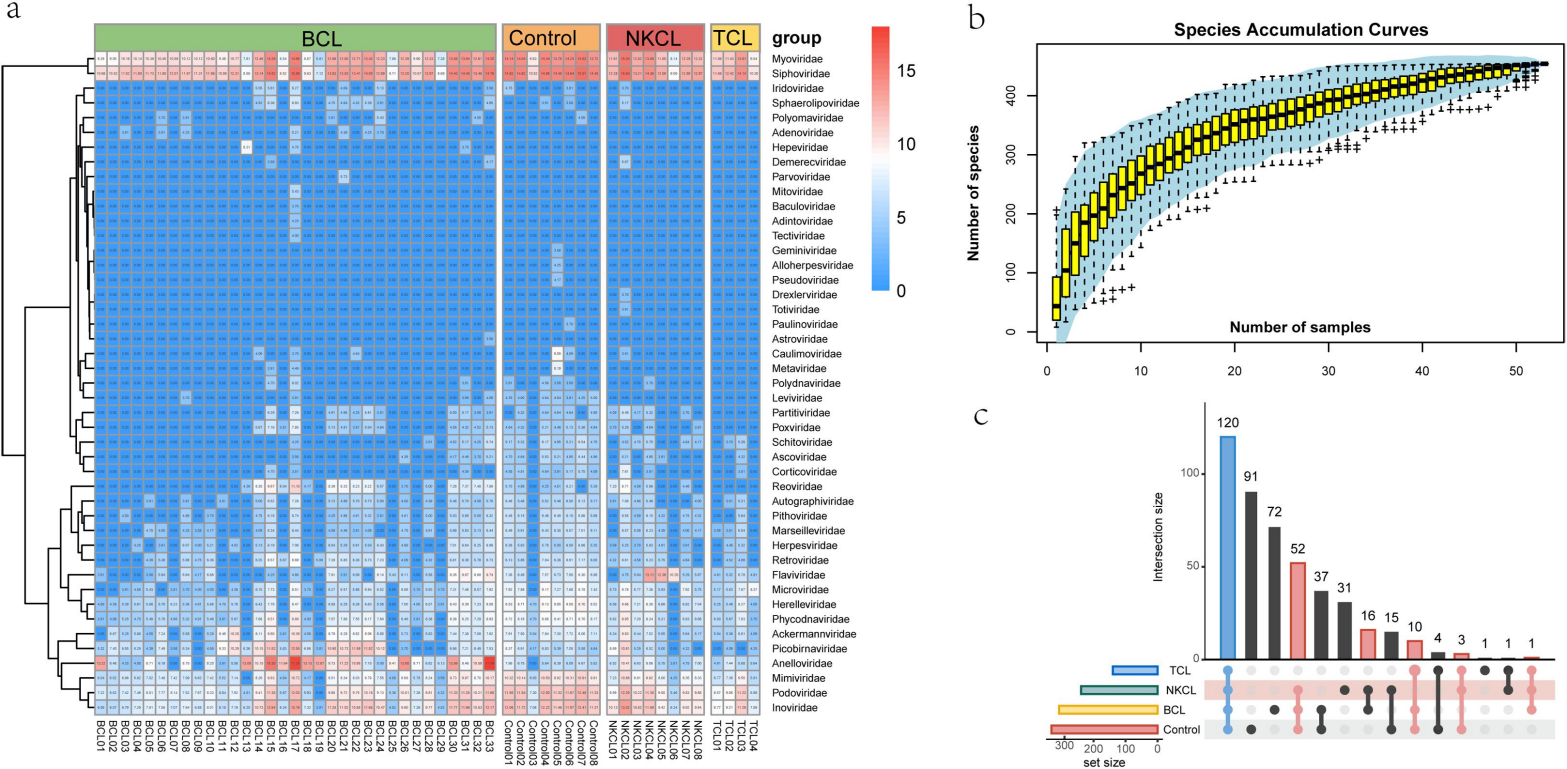
Overviewed the composition, abundance and distribution of viral communities. (a) The heat map depicted the relative abundance of viral families, where colour intensity corresponded to the level of abundance. The colour scale extended from red (indicating high abundance) to blue (indicating low abundance), as referenced by the colour bar located to the right of the heat map. The horizontal axis represented the libraries, while the vertical axis denoted the viral families. (b) The species accumulation curves of viruses in all blood samples are plotted at the species level, with the horizontal axis representing the number of libraries and the vertical axis representing the number of viral species. (c) UpSetSet plots displayed the number of shared and distinct viral species among the four groups (BCL, TCL, NKCL and healthy control). Filled coloured dots connected by vertical lines signified the intersections of shared species, whereas unfilled light grey dots denote species that do not belong to these intersections. Vertical bars represent the count of viral species within each intersection, while horizontal bars illustrated the total number of viral species in each group.

To systematically investigate compositional divergences in blood virome profiles, the 53 libraries were stratified into four cohorts: B-cell lymphoma (*n* = 33, BCL01-BCL33), T-cell lymphoma (*n* = 4, TCL01-TCL04), NK-cell lymphomas (*n* = 8, NKCL01-NKCL08), and healthy controls (*n* = 8, Control01-Control08). High-throughput sequencing through our customized bioinformatics pipeline generated 394,897 viral genomic reads (median: 7,432; range: 183–41,378 reads per library). Subsequent taxonomic classification identified 45 viral families, comprising 13 RNA virus families and 32 DNA virus families. The Hierarchical clustering analysis provided an overview of the distribution and relative abundance of viral communities across each library. It showed that, the relative abundance of *Anelloviridae* and *Picobirnaviridae* in certain libraries from the BCL group, and *Flaviviridae* in specific libraries from the NKCL group, were significantly elevated compared to other libraries (Figure 1(a)). However, no obvious differences were observed in the abundance of other viral families. Additionally, species accumulation curves reached asymptotes at ~ 400 viral species (Figure 1(b)), indicating sufficient sampling depth to characterize community diversity. Taxonomic classification of viral reads revealed 454 distinct viral species harboured in both control and patient samples. Comparative virome analysis revealed progressive diversity reduction from controls to patient groups (Control: 332 species; BCL: 308; NKCL: 239; TCL: 140). Core microbiome analysis identified 120 conserved species across all groups, with substantial overlap between controls and BCL (219 shared species). Meanwhile, the BCL and NKCL groups maintained 190 shared species. Group-specific viromic signatures were particularly evident in controls (91 unique species) and BCL (72 unique species) (Figure 1(c)).

**Figure 2.**
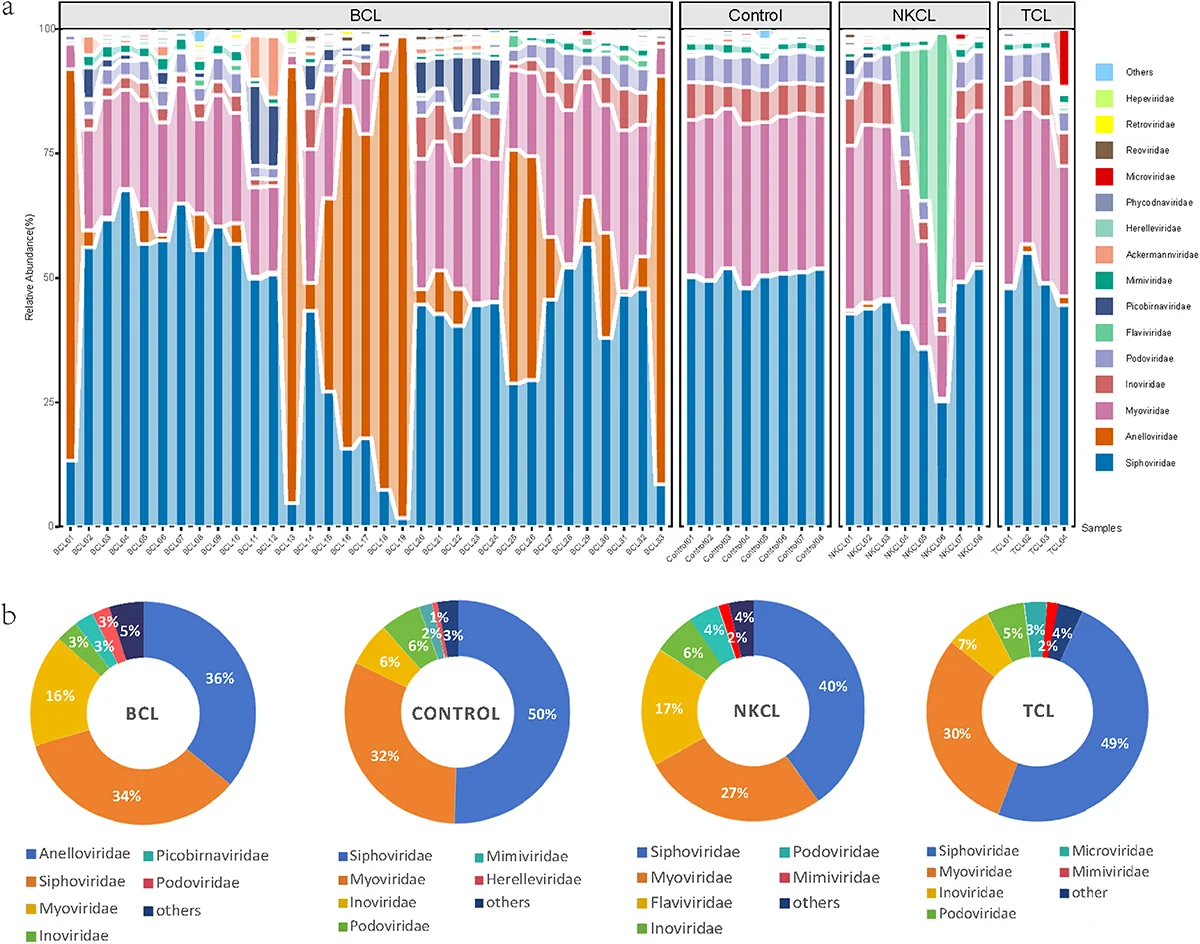
The proportion of different viral families in each library and across various groups. (a) The stacked bar chart revealed the relative abundance of different viral families in each library. (b) The doughnut chart clearly shows the proportion of each viral family in various groups.

**Figure 3.**
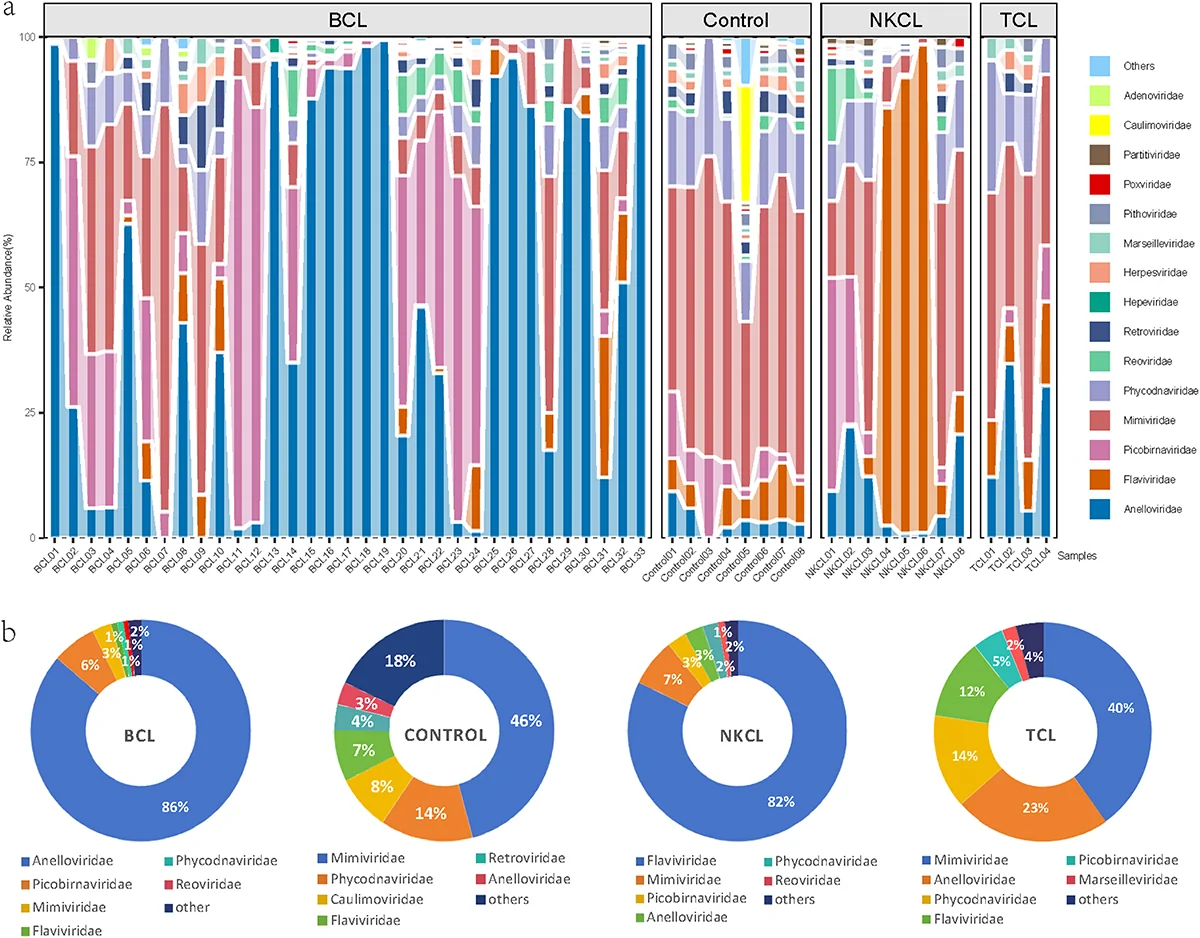
The distribution of eukaryotic viruses in each library and among different groups. (a) The stacked bar chart illustrated the relative abundance of different viral families within each library. (b) The doughnut chart vividly displayed the proportion of each viral family across four groups.

At the viral family level, bacteriophages primarily encompassing the families *Siphoviridae*, *Myoviridae*, *Inoviridae*, and *Podoviridae*, predominated in most libraries (Figure 2(a)). Control and Clinical subgroups exhibited distinct viral community structures (top 3 species): BCL: *Anelloviridae* (36%), *Siphoviridae* (34%), *Myoviridae* (16%); Control: *Siphoviridae* (40%), *Myoviridae* (27%), *Inoviridae* (17%); NKCL: *Siphoviridae* (50%), *Myoviridae* (32%), *Flaviviridae* (6%); TCL: *Siphoviridae* (49%), *Myoviridae* (30%), *Inoviridae* (7%) (Figure 2(b)). Eukaryotic virus profiling demonstrated differential taxonomic distribution patterns, *Anelloviridae* exhibited the highest abundance across all libraries, followed by *Flaviviridae*, *Picobirnaviridae*, and *Mimiviridae* (Figure 3(a)). Notably, distinct variations in viral composition were observed among different groups. Specifically, in the BCL group, *Anelloviridae* (86%), *Picobirnaviridae* (6%), *Mimiviridae* (3%), and *Flaviviridae* (1%) were the most abundant. In the NKCL group, the predominant families were *Flaviviridae* (82%), *Mimiviridae* (7%), *Picobirnaviridae* (3%), and *Anelloviridae* (3%). In the control group, *Mimiviridae* (46%), *Phycodnaviridae* (16%), *Caulimoviridae* (8%), and *Flaviviridae* (7%) were found to be the dominant viruses. Lastly, in the TCL group, *Mimiviridae* (40%), *Anelloviridae* (23%), *Phycodnaviridae* (14%), and *Flaviviridae* (12%) were the primary viral families identified (Figure 3(b)).

### Diversity analysis of the blood virome

**Figure 4.**
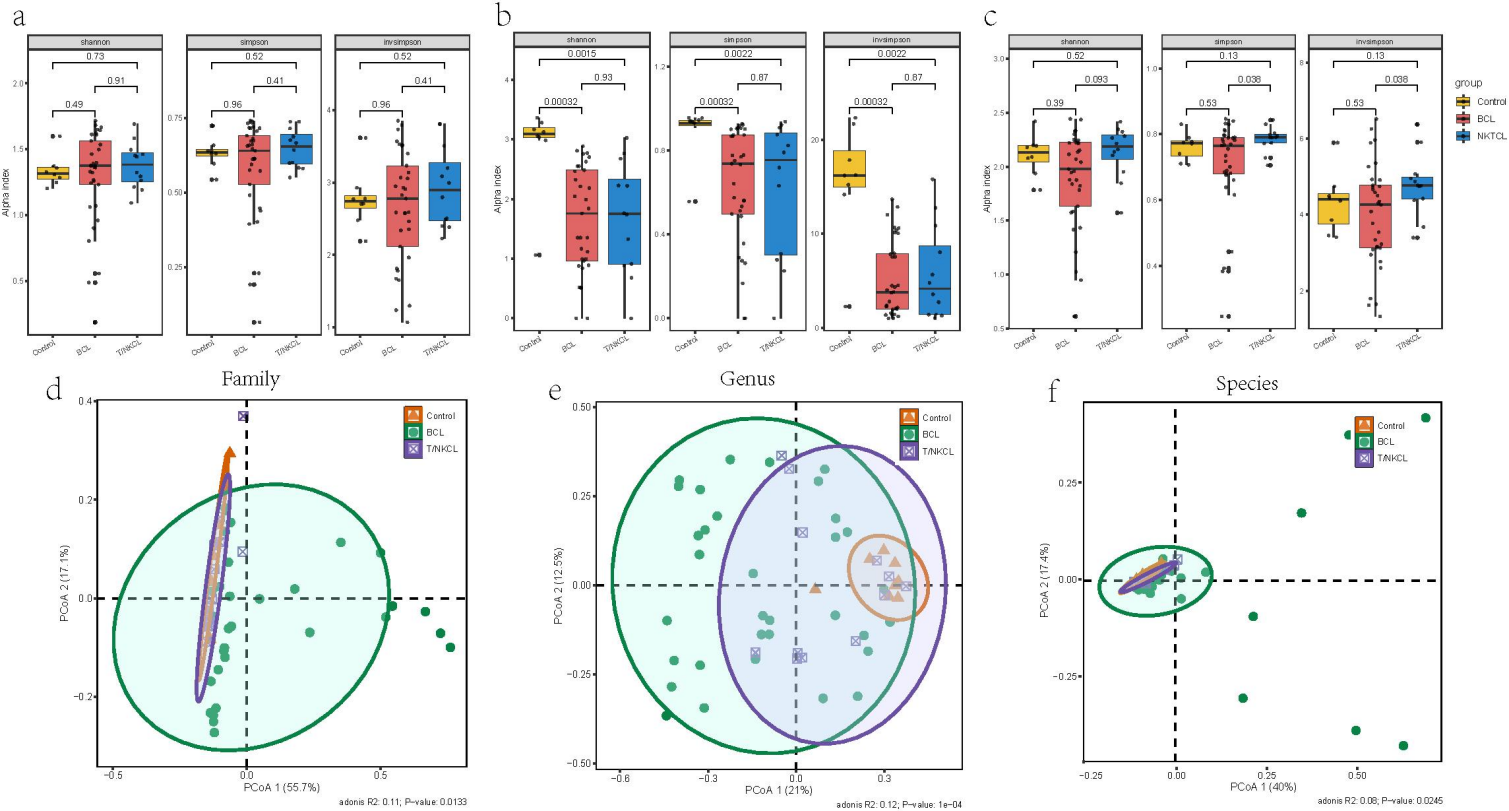
Analyses of viral diversity among B-cell lymphoma (BCL), natural killer/T-cell lymphoma (T/NKCL), and the control groups. (a–c) The alpha diversities at the family, genus, and species levels were compared among BCL, T/NKCL, and the control groups using the Shannon, Simpson, and inverse Simpson indices, respectively. The Wilcoxon test was employed to assess statistical significance, statistical significance was defined as *p* < 0.05. (d–f) Beta diversity comparisons at the family, genus, and species levels were assessed among BCL, T/NKCL, and control groups using unweighted UniFrac distances with principal coordinate analysis (PCoA). Each point, envelope and triangle represents an individual library, and the degree of separation between groups indicates their compositional dissimilarity.

Alpha and beta diversity analyses were employed to assess intra-habitat species diversity and inter-habitat compositional differences in viral communities. The experimental design comprised three comparative cohorts: BCL, T/NKCL (encompassing TCL and NKCL entities), and control cohorts. Statistical analyses were conducted using R v4.4.1 with MEGAN 6.22.2-normalized viral read data across taxonomic hierarchies (family, genus, species). Community diversity was quantified through established ecological metrics: Shannon index (H’), Simpson index (λ), and inverse Simpson index (1/λ). At the family level, both patient cohorts exhibited marginally elevated diversity indices compared to controls, though without statistical significance (Figure 4(a)). A striking reversal emerged at the genus level, where controls demonstrated statistically superior diversity metrics compared to patient groups (H:’ *p* < 0.05; λ: *p* < 0.05; 1/λ:*p* < 0.05), suggesting enhanced community richness and evenness in healthy viromes. Notably, no inter-patient group differences were observed at this taxonomic resolution (Figure 4(b)). Species-level analysis revealed comparable diversity indices between controls and patient groups. However, the T/NKCL cohort displayed significantly elevated diversity metrics relative to BCL patients (H:’ *p* = 0.093; λ: *p* < 0.05; 1/λ: *p* < 0.05), indicating distinct viral community structures between lymphoma subtypes (Figure 4(c)).

Beta diversity analysis was conducted through principal coordinate analysis (PCoA) employing unweighted UniFrac distance metrics. At the family taxonomic rank, significant β-dispersion heterogeneity was observed across cohorts, with the BCL group exhibiting substantially broader multivariate dispersion compared to both the T/NKCL and control groups (PERMANOVA: *R* = 0.11, *p* = 0.0013). In contrast, the T/NKCL and control cohorts demonstrated tightly clustered microbial community structures within comparable spatial bounds, indicating phylogenetically conserved community configurations (Figure 4(d)). Genus-level analysis revealed distinct stratification patterns: control samples displayed significantly constrained β-dispersion relative to lymphoma cohorts. While both patient groups exhibited expanded ecological heterogeneity compared to healthy controls, the BCL cohort maintained marginally greater compositional variability than the T/NKCL group (PERMANOVA: *R* = 0.12, *p* = 0.0001), suggesting subtype-specific viral community dynamics (Figure 4(e)). Notably, species-level β-diversity patterns mirrored family-level observations, with the BCL cohort again demonstrating statistically distinct clustering from other groups (PERMANOVA: *R* = 0.09, *p* = 0.0021) (Figure 4(f)). This hierarchical consistency across taxonomic resolutions implies structural differences in viral assemblages between lymphoma subtypes and healthy viromes.

### Difference of blood virome

**Figure 5.**
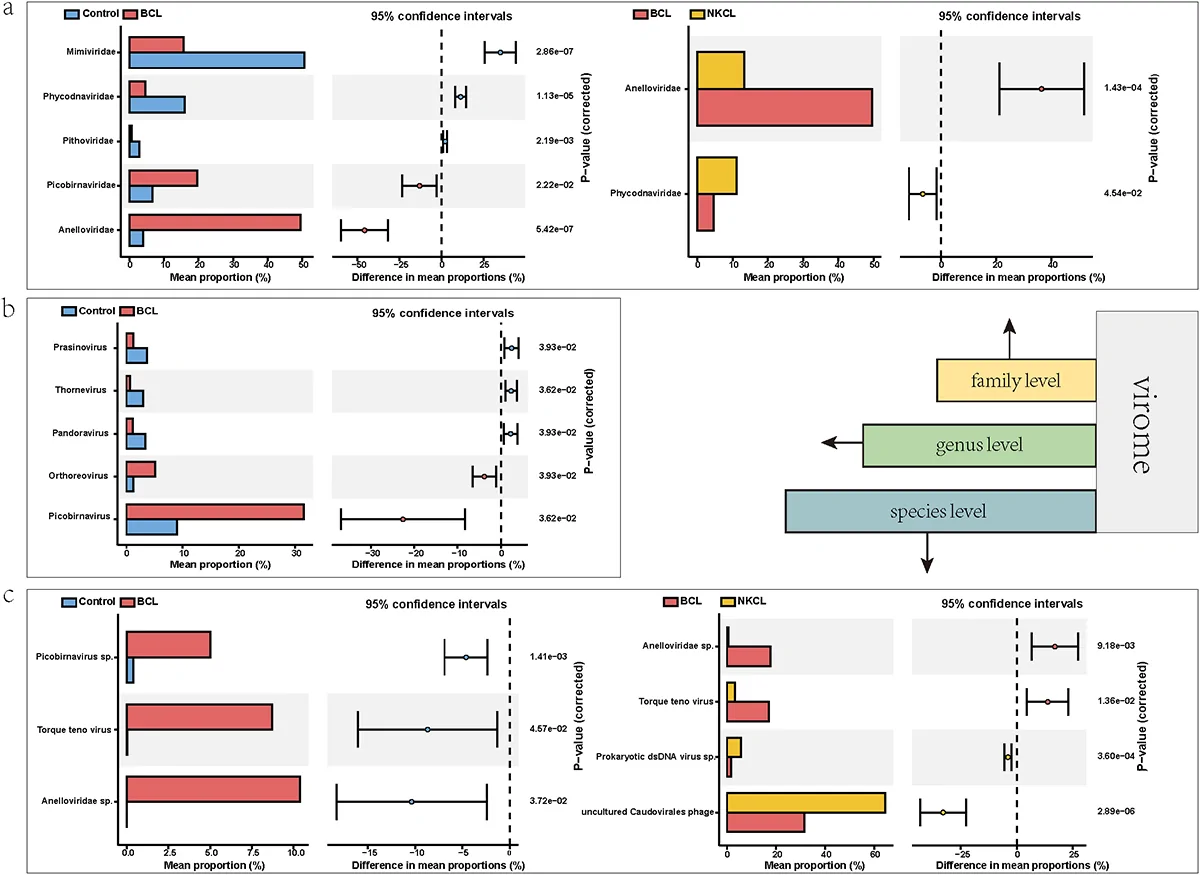
STAMP analyses of the blood virome at the family, genus and species levels. (a–c) Pairwise comparative analyses of differences in viral community relative abundance at the family, genus, and species levels were conducted among the three groups (BCL, TCL, NKCL, and healthy control) using STAMP. The bar chart in each single figure illustrated the relative abundance of specific species in different groups. Statistical analysis was performed using Welch’s t-test, with adjusted P-values less than 0.05 indicating significant differences.

Statistical Analysis of Metagenomic Profiles (STAMP) was implemented to identify differentially abundant viral taxa across taxonomic hierarchies, employing Welch’s t-test, with correction via the Benjamini-Hochberg method to control the rigorous false discovery (FDR) rate for two-group comparisons. An adjusted P-value (*P*adj) less than 0.05 is typically considered to indicate a statistically significant difference. Viral community disparities were systematically evaluated at family, genus, and species levels among defined cohorts (healthy controls, BCL and T/NKCL). Family-level analysis revealed five phylogenetically distinct viral lineages distinguishing BCL patients from controls. The BCL cohort exhibited pronounced enrichment of *Anelloviridae* (*P*adj = 5.42e-07) and *Picobirnaviridae* (*P*adj = 2.22e-02), while control samples demonstrated preferential colonization by *Mimiviridae*, *Phycodnaviridae*, and *Pithoviridae* (all *P*adj < 0.05). Inter-patient group comparisons identified *Anelloviridae* as a BCL-specific signature taxon (*P*adj = 1.43e-04) versus T/NKCL, contrasting with *Phycodnaviridae* marked difference in the latter group (*P*adj = 4.54e-02). No family-level differentiation was observed between controls and T/NKCL (Figure 5(a)). Genus-level profiling resolved five differentially abundant viral genera in BCL-control comparisons, most notably *Picobirnavirus* (*P*adj = 3.62e-02). No significant genus-level discriminators emerged in control-T/NKCL or BCL-T/NKCL contrasts (Figure 5(b)). Species-resolution analysis uncovered three BCL-associated viral signatures: *Picobirnavirus* sp. (*P*adj = 1.41e-03), torque teno virus (*P*adj = 4.57e-02), and *Anelloviridae* sp. (*P*adj = 3.72e-02) compared to the control group. Inter-patient group comparisons revealed four divergent species: BCL-enriched *Anelloviridae* sp. and torque teno virus versus T/NKCL-predominant prokaryotic dsDNA virus sp. and uncultured *Caudovirales* phage (all *P*adj < 0.05). Control-T/NKCL comparisons remained non-discriminatory at species level (Figure 5(c)).

**Figure 6.**
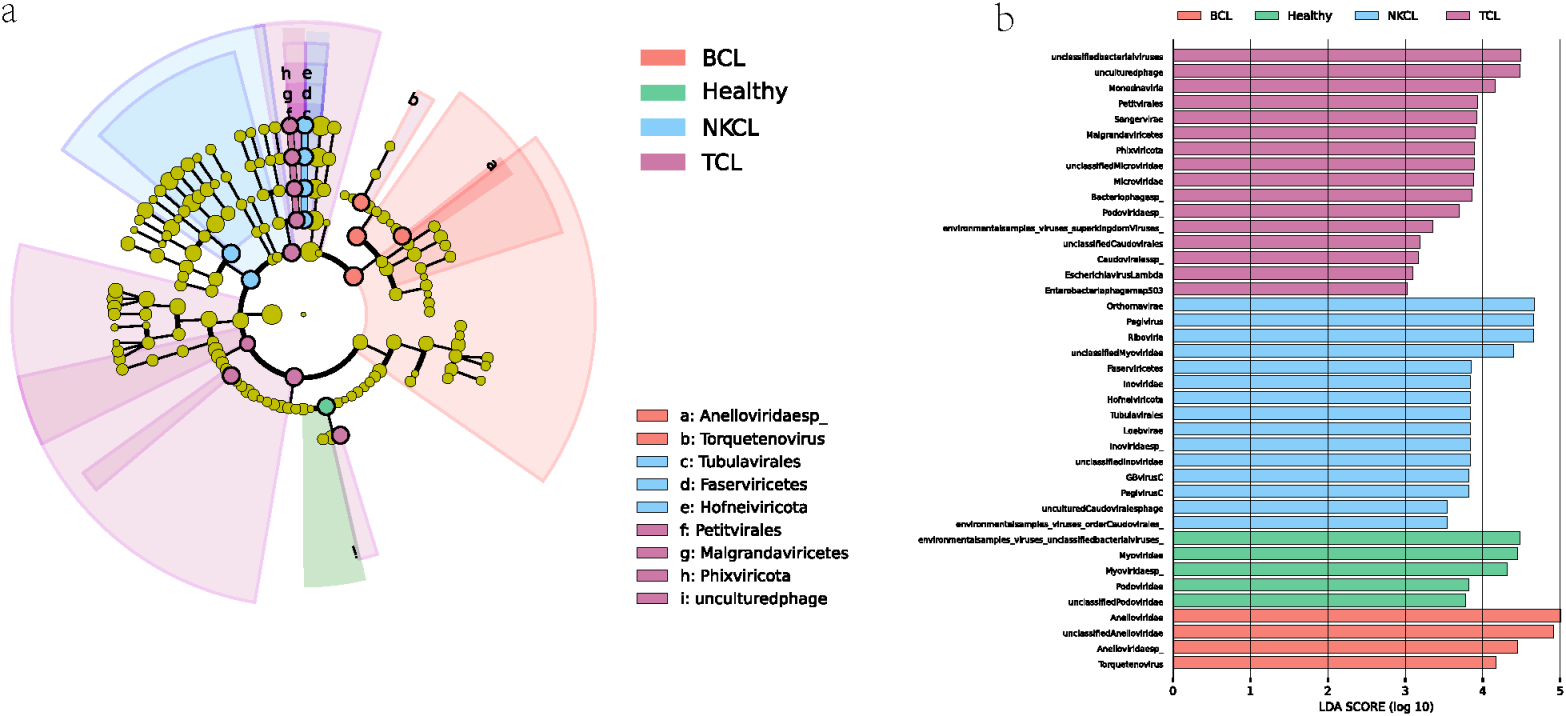
Differently abundant taxa between groups. (a) In the taxonomic cladogram, each successive circle represents a different phylogenetic level. Coloured circles highlight the groups where the specified taxa are more abundant compared to other groups, whereas yellow circles signify no significant difference. The differing taxa are displayed on the right side of the cladogram. (b) Linear discriminant analysis. The taxa with the most significant differences in abundance are depicted in a bar graph based on their LDA scores (log10), which estimate the effect size. Only those taxa with a P-value < 0.05 and a LDA score exceeding the significance threshold of |3| are displayed.

Potential biomarker Linear identification were conducted using Linear Discriminant Analysis Effect Size (LEfSe) with Kruskal-Wallistest and Wilcoxon test. The blood virome in the BCL group was characterized by a preponderance of *Anelloviridae* (including Torque teno virus species, unclassified *Anelloviridae*, and *Anelloviridae* sp.), consistent with the results of STAM analysis. In contrast, *Picobirnaviridae*, along with its genus and species, were not identified, although they were detected in the STAM analysis. In the NKCL group, the genus of pegivirus and the species pegivirus-C (HPgV-1) were delineated. However, in the control group and TCL group, no differential eukaryotic viruses infecting vertebrates were detected, except for bacteriophages (Figure 6(a,b)).

### Identification and analysis of pegivirus and anelloviruses

**Figure 7.**
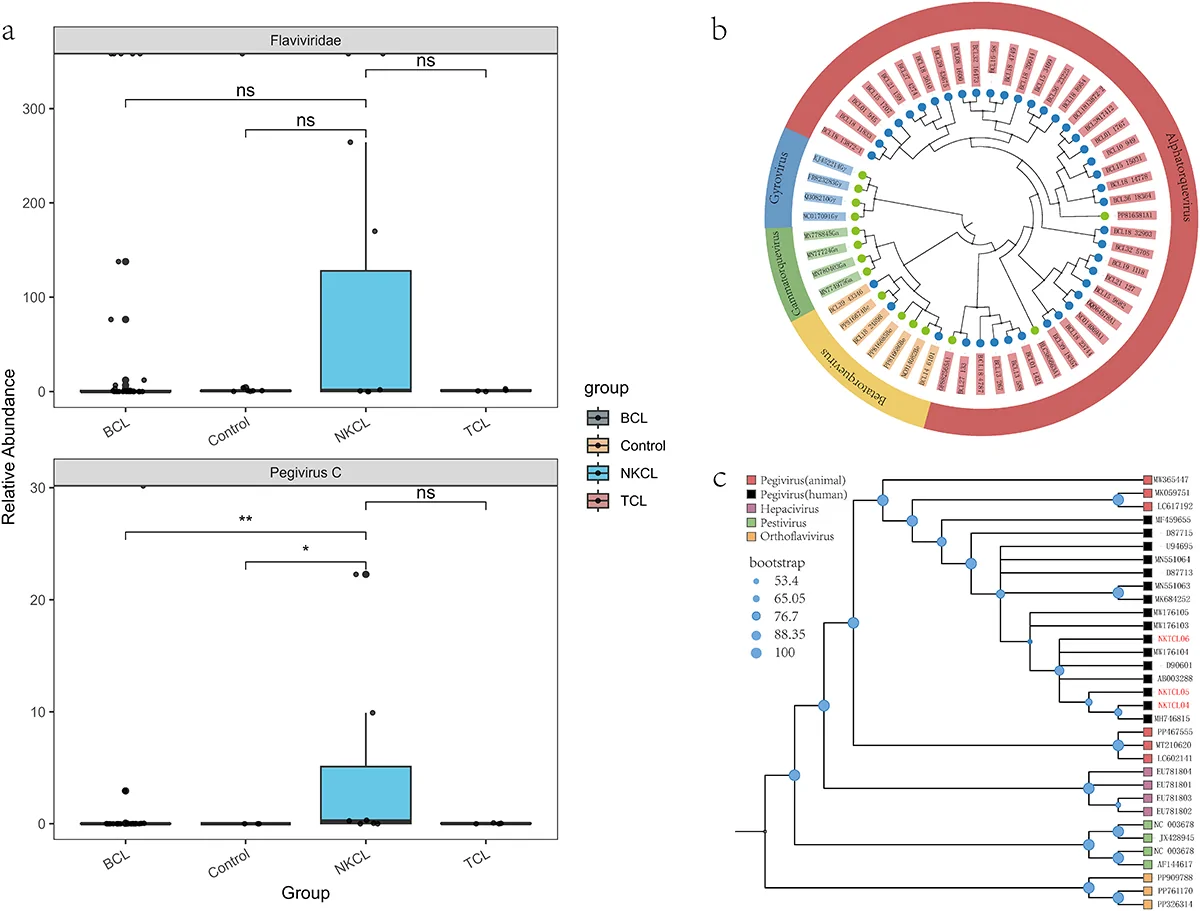
The identification and phylogenies of human pegiviruses and anelloviruses identified in the blood of non-Hodgkin lymphoma patients. (a) The differences in relative abundance of flaviviridae and HpgV-1 between each pair of the four groups. (b) An ML phylogenetic tree of anelloviruses was constructed based on the amino acid sequences of ORF1. In this study, ORF1 sequences were marked with blue dots at each node, whereas reference sequences were indicated with green dots. (c) Maximum likelihood (ML) phylogenetic tree was constructed based on the polyprotein sequences of pegiviruses and reference sequences spanning multiple genera of flaviviridae using MEGA v11.0.13. The names of the pegiviruses identified in this study were marked with red.

Human pegivirus (HPgV-1), a member of the *Flaviviridae* family and first identified as GB virus C (GBV-C), is a positive-sense single-stranded RNA virus with a genome of 9.4 kb in length [24]. HPgV includes type 1 (HPgV-1) and type 2 (HPgV-2), both with genomes organized similarly to that of hepatitis C virus (HCV) [25,26]. Although HPgV-1 can cause persistent infection, it does not cause the occurrence of related diseases in healthy people [27]. It globally infects humans, primarily through blood and body fluids, with high prevalence in immunocompromised populations, such as HIV and/or HCV positive individuals [28,29]. However, emerging evidence suggests a potential epidemiological link to lymphomagenesis. Studies have reported increased pegivirus detection in BCL, particularly diffuse large B-cell lymphoma (DLBCL), compared to healthy controls [30,31]. In this study, although no significant differences in the abundance of *Flaviviridae* and HPgV-1 were found among T/NKCL, BCL, and the control groups in STAMP analysis, significant differences in the abundance of HPgV-1 were identified between BCL and NKCL, and between Control and NKCL using the Wilcoxon paired test, which is in agreement with the LEfSe analysis results (Figure 7(a)). Furthermore, we obtained three nearly complete coding sequences (CDS) encoding the polyprotein of HPgV-1 from the NKCL group. These sequences have lengths of 8,450 bp, 8,889 bp, and 8,817 bp from the libraries of NKCL04, NKCL05, and NKCL06, respectively.

Phylogenetic analysis based on the polyprotein sequences of pegiviruses and reference sequences spanning multiple genera of *Flaviviridae* indicated that they cluster with human pegivirus-1 and showed over 95% similarity to their best match (Figure 7(c)). To investigate pegivirus prevalence in the sequencing libraries, raw data from each library were aligned against two reference pegivirus genomes (GenBank accession numbers MH746815 and LZ229595) using the nucleotide mapping algorithm implemented in Geneious Prime v2024.0.5 (Biomatters Ltd). This analysis employed default alignment parameters (medium sensitivity, 5 iterations) to maximize detection sensitivity while maintaining sequence specificity. The results showed 8 of 33 libraries were positive for HPgV-1 in the BCL group, 7 of 8 libraries were positive in the NKCL group, 3 of 4 libraries were positive for HPgV-1 in the TCL group, while in the healthy group all the 5 libraries were negative for HPgV-1.

The *Anelloviridae* family comprises small circular single-stranded DNA viruses (1.6–3.9 kb) characterized by non-enveloped capsids and ambisense genomic organization [32]. Current ICTV taxonomy (2023 release) recognizes 33 established genera containing 173 species, differentiated through phylogenetic divergence of the conserved ORF1-encoded capsid protein. To date four human infecting genera have been discovered: Alphatorquevirus (31 species), Betatorquevirus (38 species), Gammatorquevirus (15 species), and Hetorquevirus (8 species) [33]. While *Anelloviridae* infections often result in lifelong viraemia without overt clinical symptoms, they have been implicated as cofactors in disease progression, particularly in immunocompromised individuals [34]. For instance, high viral loads of *Anelloviridae* have been associated with various cancers, autoimmune diseases, and viral infections [35–37]. Through systematic computational screening of sequence repositories, we identified 38 anelloviruses, and each of which containing an intact ORF1 domains were phylogenetically validated. Phylogenetic analysis revealed that 3 anelloviruses were clustered within the Betatorquevirus genus, while the remaining 35 anelloviruses were grouped into the Alphatorquevirus genus (Figure 7(b)).

## Discussion

To our knowledge, the present study provides the first comprehensive characterization of the blood virome in NHL patients through viral metagenomic analysis, revealing distinct viromic signatures associated with lymphoma subtypes and suggesting potential viral contributions to lymphomagenesis. Our findings demonstrate compositional shifts in the blood virome between NHL patients and healthy controls, particularly marked by the enrichment of *Anelloviridae* in BCL and HPgV-1 in NKCL. These observations are consistent with emerging evidence suggesting that NHL is often associated with chronic viral infections, which contribute to the pathogenesis of lymphoid malignancies through immune dysregulation and oncogenic mechanisms [38–40].

The pronounced expansion of Anelloviridae in BCL patients (86% of total eukaryotic viral abundance compared to 3% in controls) aligns with our previous research on lymphoma [41] and further supports findings that link torque teno virus (TTV) load to immunosuppression and B-cell dysfunction [34]. The phylogenetic clustering of identified *Anelloviridae* within Alphatorquevirus and Betatorquevirus genera known to exhibit persistent viraemia in immunocompromised hosts [42] suggests their potential role as biomarkers of immune status in lymphoma. The inverse correlation between *Anelloviridae* abundance and alpha diversity at genus level may reflect viral dominance over commensal virome components during lymphomagenesis. This ecological shift parallels observations in HIV-associated lymphomas, where *Anelloviridae* expansion correlates with CD4+ T-cell depletion. This expansion also leads to decreased abundance of some viruses and reduced diversity of the microbiome [43]. However, mechanistic studies are needed to establish causality in NHL.

The NKCL-specific predominance of *Flaviviridae* (82% abundance), particularly HPgV-1, presents a novel association requiring further investigation. While HPgV-1 viraemia is associated with lymphoma risk, overall and for the major lymphoma subtypes, including diffuse large B-cell, follicular, marginal zone, and T-cell lymphomas [30], it does not mention NKCL. The striking prevalence of HPgV-1 in NKCL libraries (7/8 positive) compared to healthy controls (0/8) suggests subtype-specific tropism. The phylogenetic proximity of identified pegivirus strains to HCV-related viruses raises questions about shared oncogenic pathways, given HCV’s established role in marginal zone lymphoma via chronic antigen stimulation [44]. Notably, the absence of HPgV-1 in controls contrasts with its reported 0.8–46.6% prevalence in healthy populations in the developing world [24], possibly indicating enhanced viral replication or impaired clearance in lymphoma patients.

The reduced virome diversity observed in NHL patients across taxonomic levels (species richness: controls = 332 vs. TCL = 140) aligns with the “virome depletion” hypothesis observed in solid tumours [45], potentially reflecting immune-editing of commensal viruses during malignant transformation. The β-diversity patterns revealed through PCoA particularly the expanded dispersion in BCL viromes indicate greater inter-patient heterogeneity in viral community structures, possibly mirroring the molecular diversity of B-cell lymphomas [46]. Conversely, the conserved viromic architecture in T/NKCL patients may reflect shared etiopathogenetic mechanisms, such as EBV co-infection patterns in NK/T-cell malignancies [47].

Phages are viruses that specifically infect bacteria and are incapable of infecting eukaryotic cells. Despite this, a small proportion of phages can traverse epithelial cell layers and disseminate within the traditionally sterile regions of the human body, such as the blood, lymph, organs, and even the brain [48]. In this study, phages were found to be the dominant viral entities in both patient and healthy control cohorts. Additionally, the presence of certain algal and plant viruses (*Phycodnaviridae* and *Partitiviridae*) were detected, indicating potential contamination of the samples or viral libraries by phages and algal viruses during sample collection or library construction.

Methodologically, the pooling strategy (4–5 samples per library) introduced limitations in resolving individual-level viromic variations, potentially masking rare viral taxa. Furthermore, the cross-sectional design precludes causal inference regarding virome alterations preceding versus resulting from lymphoma development. Longitudinal studies tracking viromic dynamics during disease progression and treatment are warranted to establish temporal associations.

Clinically, the identification of subtype-specific viral signatures (*Anelloviridae* in BCL, HPgV-1 in NKCL) highlights the potential for viromic profiling as a diagnostic adjunct. *Picobirnaviridae* has been implicated in causing diarrhoea, with a higher detection rate in faecal samples from immunocompromised individuals and children with diarrhoea [49]. Compared with the healthy control, NKCL, and TCL groups, the significant enrichment of Picobirnaviridae observed in patients with BCL may be attributed to the compromised immune status of these patients, which could predispose them to an increased likelihood of viral infection. Future research should prioritize functional validation of virome-host interactions, particularly *Anelloviridae*-mediated modulation of Toll-like receptor pathways and HPgV-1 effects on NK-cell function, which may elucidate mechanistic links to lymphomagenesis.

In conclusion, this study establishes the blood virome as a dynamic ecosystem exhibiting NHL subtype-specific alterations. The identified viral signatures not only deepen our understanding of lymphoma biology but also open avenues for developing virome-targeted diagnostic tools and immunomodulatory therapies. Further integration of viromic data with host transcriptomic and immunophenotypic profiles will be critical for translating these findings into clinical applications.

## List of Αbbreviation

**Abbreviation**: **Full Name**
HL: Hodgkin lymphoma
NHL: Non-Hodgkin lymphoma
BCL: B-cell lymphoma group (all non-Hodgkin lymphomas of B-cell origin, WHO-HAEM5 Level 1 Category “Mature B-cell neoplasms”)
TCL: T-cell lymphoma group (all non-Hodgkin lymphomas of T-cell origin, WHO-HAEM5 Level 1 Category “Mature T-cell and NK-cell neoplasms”)
NKCL: NK-cell lymphoma group (all non-Hodgkin lymphomas of natural killer – cell origin, WHO-HAEM5 Level 1 Category “Mature T-cell and NK-cell neoplasms”)
T/NKCL: Combined T-cell and NK-cell lymphoma group (TCL+NKCL)
STAMP: Statistical Analysis of Metagenomic Profiles
LEfSe: Linear Discriminant Analysis Effect Size
HPgV-1: Human pegivirus-1
Padj: adjusted P-value
PCoA: Principal coordinate analysis
PERMANOVA: Permutational Multivariate Analysis of Variance
STAMP: Statistical Analysis of Metagenomic Profiles

## Data Availability

The raw sequence reads generated by Illumina in the study are available at the NCBI Sequence Read Archive database under the BioProject accession PRJNA1215684 (https://www.ncbi.nlm.nih.gov/bioproject/?term = PRJNA1215684) and PRJNA1195412 (https://www.ncbi.nlm.nih.gov/bioproject/?term = PRJNA1195412). All anellovirus and pegivirus sequences identified in this study have been deposited in the GenBank database under the accession numbers ranging from PV113253 to PV113253. Detailed information is listed in Supplementary Table S4. All Supplementary Tables (S1-S4) have been deposited in Figshare and are publicly available. The supplementary data can be accessed via the following DOI: https://doi.org/10.6084/m9.figshare.28681427.

## What’s new

This study pioneers the characterization of blood virome alterations in Non-Hodgkin lymphoma (NHL), revealing subtype-specific signatures: *Anelloviridae* dominates B-cell lymphoma (86% vs. 3% controls), linking to immunosuppression, while NK-cell lymphoma shows Flaviviridae enrichment (82%), primarily HPgV-1. Compared with healthy controls, patients with NHL exhibited significantly lower blood virome α-diversity at the genus level, and T-cell lymphomas showed the lowest species-level richness. These findings identify viral biomarkers for NHL subtyping and potential therapeutic targets, advancing understanding of virome-lymphomagenesis interactions.
